# Emergency department patient‐centred care perspectives from deaf and hard‐of‐hearing patients

**DOI:** 10.1111/hex.13842

**Published:** 2023-08-09

**Authors:** Tyler G. James, Meagan K. Sullivan, Michael M. McKee, Jason Rotoli, David Maruca, Rosemarie Stachowiak, JeeWon Cheong, Julia R. Varnes

**Affiliations:** ^1^ Department of Family Medicine University of Michigan Ann Arbor Michigan USA; ^2^ Department of Health Education and Behavior University of Florida Gainesville Florida USA; ^3^ Department of Psychiatry University of Florida Gainesville Florida USA; ^4^ Department of Emergency Medicine University of Rochester Medical Center Rochester New York USA; ^5^ Unaffiliated Researcher Florida USA; ^6^ Department of Health Services Research, Management, and Policy University of Florida Gainesville Florida USA

**Keywords:** deaf and hard of hearing, emergency medicine, healthcare seeking, patient‐centred care, patient–provider communication

## Abstract

**Background:**

Deaf and hard‐of‐hearing (DHH) patients are a priority population for emergency medicine health services research. DHH patients are at higher risk than non‐DHH patients of using the emergency department (ED), have longer lengths of stay in the ED and report poor patient–provider communication. This qualitative study aimed to describe ED care‐seeking and patient‐centred care perspectives among DHH patients.

**Methods:**

This qualitative study is the second phase of a mixed‐methods study. The goal of this study was to further explain quantitative findings related to ED outcomes among DHH and non‐DHH patients. We conducted semistructured interviews with 4 DHH American Sign Language (ASL)‐users and 6 DHH English speakers from North Central Florida. Interviews were transcribed and analysed using a descriptive qualitative approach.

**Results:**

Two themes were developed: (1) DHH patients engage in a complex decision‐making process to determine ED utilization and (2) patient‐centred ED care differs between DHH ASL‐users and DHH English speakers. The first theme describes the social‐behavioural processes through which DHH patients assess their need to use the ED. The second theme focuses on the social environment within the ED: patients feeling stereotyped, involvement in the care process, pain communication, receipt of accommodations and discharge processes.

**Conclusions:**

This study underscores the importance of better understanding, and intervening in, DHH patient ED care‐seeking and care delivery to improve patient outcomes. Like other studies, this study also finds that DHH patients are not a monolithic group and language status is an equity‐relevant indicator. We also discuss recommendations for emergency medicine.

**Patient or Public Contribution:**

This study convened a community advisory group made up of four DHH people to assist in developing research questions, data collection tools and validation of the analysis and interpretation of data. Community advisory group members who were interested in co‐authorship are listed in the byline, with others in the acknowledgements. In addition, several academic‐based co‐authors are also deaf or hard of hearing.

## 1 INTRODUCTION

Inaccessible communication in all settings can negatively impact the mental health, physical health and quality of life of people who are deaf and hard of hearing (DHH).^1^, ^2^, ^3^, ^4^, ^5^, ^6^, ^7^, ^8^, ^9^, ^10^, ^11^ In the United States, 49–55 million people have hearing loss,^12^, ^13^, ^14^ and DHH people with younger ages of onset are at higher risk of poor familial communication, which is linked to poorer health outcomes in adulthood.^10^, ^15^ Federal regulations in the United States (e.g., Section 504 of the Rehabilitation Act of 1973, the Americans with Disabilities Act and the Patient Protection and Affordable Care Act) protect DHH patients' communication access but, despite these regulations, many healthcare facilities are noncompliant.^16^, ^17^, ^18^ Consequently, DHH patients report ineffective communication in healthcare environments,^6^, ^19^, ^20^ including the emergency department (ED).^21^

DHH people in the United States primarily communicate through American Sign Language (ASL) or spoken language. DHH people who use spoken language typically have older ages of onset (e.g., age‐related hearing loss), providing a strong language base and fund of information. DHH ASL‐users are more likely to have congenital or childhood ages of onset and, due to poor communication access during childhood,^2^ may have limited English proficiency,^22^ poorer health literacy^23^, ^24^ and limited access to consistent health education and health‐promoting resources.^11^, ^25^ Despite these differences, DHH people—collectively and when segmented by language status—are at higher risk of ED utilization than people who are not DHH.^26^, ^27^ For example, DHH ASL‐users have higher adjusted prevalence ratios of using the ED than non‐DHH English speakers.

There have been several quantitative studies of ED utilization and care processes among people who are DHH.^26^, ^27^, ^28^, ^29^, ^30^, ^31^ Despite the socioeconomic differences among DHH people, this population is at higher risk than non‐DHH English speakers of using the ED,^26^, ^27^ and DHH ASL‐users are at higher risk of longer ED length of stay (LOS).^28^, ^30^ Despite the growing quantitative literature in this area, there is a lack of qualitative research focused on ED care for DHH people.^26^, ^32^ Qualitative studies—among patients, providers and other stakeholders—will enable health services researchers and ED providers to better understand patient‐ and nonpatient factors impacting ED utilization and care for DHH patients. Therefore, the purpose of this study was to qualitatively assess DHH patients' experiences with seeking ED care and how care was provided in the ED.

## 2 METHODS

### 2.1 Theoretical framework

We applied the *Conceptual Model of ED Utilization among DHH Patients* ^11^ throughout the study. This model represents empirical and theoretical findings related to DHH patient ED utilization and care delivery. Consistent with the assumptions of the conceptual model, the authors applied the transformative paradigm of research.^33^, ^34^, ^35^ Methodologically, this conceptual framework and paradigm informed all research aims. For example, in this study, we sought to apply transformative sampling considerations to identify patients who are multiply marginalized.^36^ We also applied the Critical Disability and DeafCrit^37^ lens when analysing data, interrogating the role of audism and intersectional oppression.

### 2.2 Study design

This study was the qualitative phase of an explanatory sequential mixed‐methods dissertation study. In this design, quantitative results inform the development of qualitative data collection procedures to further explain quantitative findings.^38^ Mixed‐methods integration procedures included *connecting* and *building*.^39^, ^40^ Specifically, we developed purposeful sampling criteria to best capture and explain quantitative findings; in addition, the interview guide was developed using quantitative results. Quantitative findings^26^, ^28^ and methodological innovations^36^ from this study are published elsewhere. We will explain and contextualize our previous quantitative findings by exploring participants' experiences using qualitative descriptions.^41^, ^42^ All procedures were approved by the University of Florida's Institutional Review Board (IRB#201903489). The funding sponsors had no role in the conduct of this study.

### 2.3 Setting

The study was conducted in north central Florida, focusing on patients who used the ED of a large academic medical centre. The academic medical centre includes one hospital‐based ED and two freestanding, full‐service EDs. The hospital is a designated Level 1 Trauma Center. There are no on‐staff ASL/English interpreters in the medical centre; instead, the health system contracts with community‐based agencies and provides web‐based video remote interpreting (VRI) services.^21^ This process for providing interpreters to DHH ASL‐users is consistent with contexts across the United States.^17^, ^18^, ^43^

### 2.4 Sample and recruitment

We sought sufficient depth of information to draw credible conclusions based on how the data were analysed.^44^, ^45^, ^46^, ^47^ Guided by concepts related to information power in qualitative research,^46^ community advisory board recommendations, previous work with this population^20^, ^21^, ^32^ and resources, we aimed to interview 10 DHH ASL‐users and 10 DHH English speakers. Purposeful criterion sampling prioritized recruiting DHH people who were Black; from rural areas; believed that substance use was an issue in their community and who were diverse with respect to DHH identities (e.g., late‐deafened or DeafBlind),^36^ as these populations are less represented in DHH health services research and experience unique social circumstances. This was in addition to eligibility criteria: identifying as a DHH person or person with hearing loss, who was 18 years or older and who had been a patient in the academic medical centre's EDs within 36 months before the interview.

Recruitment took place through referral from community advisory board members, advertisements through partner organizations and social media. To better achieve purposeful sampling goals and to decrease the impact of the COVID‐19 pandemic, we partnered with a non‐DHH‐focused community‐based research referral programme^48^ to recruit DHH English speakers. Despite the priority on marginalized communities of DHH people, the research referral programme changed prioritized criteria without permission from the research team; this change was not identified until after participants initiated participation in the study. Therefore, the sample is less diverse than intended with respect to socioeconomic position, neighbourhood characteristics and race. The sample, however, is diverse with respect to DHH‐specific characteristics (Table 1). (The deviation from the sampling plan was reported to the IRB and investigator team).

**Table 1:** Deaf and hard‐of‐hearing interviewee characteristics.

Characteristic	ASL‐Users (*N* = 4)	English speakers (*N* = 6)	Total (*N* = 10)
Age, years			
18–44	3	1	4
45–64	1	2	3
65+	0	3	3
Gender			
Man	1	1	2
Woman	3	6	9
Race/ethnicity			
White	3	5	8
Black/African American	1	0	1
Hispanic	0	1	1
Education			
Did not graduate high school	0	1	1
High school diploma	1	0	1
Some college	2	1	3
College graduate, Bachelor's	1	4	5
Age of onset			
Before the age of 3 years	4	1	5
Between 3 and 19 years of age	0	2	2
After 19 years of age	0	3	3
Language preference—everyday			
Sign language	2	0	2
Sign language + English	2	0	2
English	0	5	5
English + Spanish	0	1	1
Language preference—healthcare			
Sign language	3	0	3
Sign language + English	1	0	1
English	0	6	6
ED use, past 3 years			
Range	3 to >10	1 to >10	1 to >10
Interview modality and length			
Zoom	4	2	6
Telephone	0	4	4
Length of interviews, range	46–60 min	23–89 min	23–89 min
Length of interviews, median	49 min	58 min	54 min

Abbreviations: ASL, American Sign Language; ED, emergency department.

After participants were screened and found to be eligible, they received informed consent information and were scheduled for a web‐based video or telephone‐based audio‐only interview. Participants were provided an incentive: $40 for the first interview and $35 for the follow‐up member check interview. All interviews were conducted by the first author, a non‐DHH, English‐ and ASL‐fluent researcher with 7 years of engagement with the local DHH community at the time of data collection. Interviews were conducted between December 2020 and June 2021.

### 2.5 Data collection

The data sources for this study were from primary interviews with DHH patients, member check interviews with patients and community advisory board members and the interviewer's reflective and analytic memos. The interview guide (see Supporting Information: Material 1) was developed in partnership with the community advisory board and guided by the conceptual model and quantitative results. Interviews were recorded and conducted in the preferred language of the participant (i.e., spoken English or ASL). Interviews in English were professionally transcribed; interviews in ASL were translated and transcribed into English by a certified deaf interpreter (D. M.) with almost a decade of interpreting experience.

Member check (follow‐up) interviews were used to receive feedback from participants and community advisory board members on interim results.^49^ We asked participants to identify if the synthesis of their experience accurately represented their lived experience; answer clarifying questions and provide additional insight, particularly if they had used the ED in the time since the initial interview. Member check interviews were not recorded or transcribed; instead, memos and direct quotes were entered simultaneously into the data analysis software.

### 2.6 Data analysis

The interviewer coded all data on the DHH English speakers' transcripts and on the DHH ASL‐users' videos using MAXQDA (Verbi Software, 2021). The interviewer coded constructs from the conceptual model through content analysis. While conducting this coding, he made memos and developed inductive codes.^50^ Coding decisions were discussed with a member of the community advisory group, who provided feedback on coded insights and memo descriptions. Themes were developed by collapsing codes into specific categories through a process of codeweaving. Codeweaving is the ‘integration of key word codes and phrases into narrative form to see how the puzzle pieces fit together’.^50^ We also used code relation frequencies and interactive code matrices in MAXQDA, in addition to the ‘touch test’^50^ and peer debriefing.^45^

## 3 RESULTS

### 3.1 Theme 1: DHH patients engage in a complex decision‐making process to determine ED utilization

#### 3.1.1 Evaluating need

Evaluating need describes the process through which DHH patients assess their need to use healthcare after experiencing symptoms of an acute injury or condition, acute‐on‐chronic presentation or symptoms of chronic health issues. When evaluating need, participants first described comparing their symptoms with their knowledge of their medical history and other health conditions that they may be experiencing.

I was having some sharp pain in my lower left abdomen, almost down by the crease in the leg. And it'd been getting worse over several days and I'm thinking, ‘Well, the only organ down there is the intestines and the skin. There are no other organs there’. It didn't clear up. At one point in the morning, I emptied my bowels and they were black and tarry (i.e., blood). (English speaker)

These participants' consideration of their understanding of anatomy and their approach to wait to see if the issue was self‐resolving led them to ‘immediately’ seek ED care after they felt that their symptoms worsened. Other participants sought additional self‐diagnostics to assist with confirming their initial suspicions.

We have an at‐home blood pressure kit…I don't use it as part of my daily routine, I only use it when I'm questioning how I'm feeling…The heart rate was 160. The blood pressure was over 200. So, you know, that sort of raised a red flag to have my heart rate at 160 and not coming down. (English speaker)

Past experiences of using healthcare and the ED also manifested in DHH participants' evaluation of need, including previous encouragement from providers to use the ED (when experiencing a specific symptom) or if the patient knew that the issue was related to chronic health issues. For example, during pregnancy, a DHH English speaker was told to seek ED care if they ever felt that the foetus was less active; this provider's guidance led to multiple ED visits when this symptom occurred.

For some participants, however, individual health literacy and past experience were insufficient for them to immediately determine their needs. These participants then sought additional information from other sources.

I looked it up online…I was mostly looking at different symptoms and how they pair with potential diagnoses. Which diagnosis appears with which symptoms, and which fit my condition. After enough searching and finding enough commonalities, I knew what I was dealing with. (ASL‐user)

Also, while some participants discussed their concerns with friends and family members, others evaluated their need to seek ED care by themselves. Those who sought social network feedback, however, indicated that the conversations affirmed their decisions to go to the ED.

#### 3.1.2 Care‐seeking decisions

After patients evaluated their need to use healthcare based on their symptoms and condition, patients then went through a decision‐making process to wait and see; contact nurse triage or primary care; go to urgent care; call 911 or go immediately to the ED. These categories are described and displayed with quotes in Table 2.

**Table 2:** Categories and quotes related to ED care‐seeking decisions among DHH patients.

Category	Description	Quote(s)
Wait and see	Participants determined that seeking ED care was not an immediate need. Participants engaged in self‐monitoring or self‐treatment and re‐evaluation.	My headache was getting worse and worse. It wasn't like my normal headaches. I took Aleve or Advil, which typically makes my headaches go away. It didn't this time, so I decided to go to the ER. (ASL‐user) My arm was hurting badly, but I tried to push it out for as long as possible so I could go to my primary care doctor instead of the ER. But the pain was getting worse, and since it was the weekend, I couldn't see my doctor. (ASL‐user)
Contact nurse triage or primary care	Participants sought a limited evaluation of their symptoms through the nurse triage line provided by their health insurance plans or contacted their primary care providers. Multiple barriers were reported for engaging in ambulatory care: limited schedule availability, changes due to the COVID‐19 pandemic, length of time to get a response.	When this happened before, I called nurse triage. They said to go to the ER then. Then, the ER people said, ‘If this happens again, just go directly to the ER. Don't bother to call nurse triage. (English speaker) Since COVID happened, my doctor's office doesn't let us go into the office anymore. They've been requiring us to answer screening questions through Zoom or the telephone. I have to go to a COVID testing site…that delays the process. It's not worth it, so I go to the emergency room because it is faster – I can get everything in one day. (ASL‐user)
Go to urgent care	Several participants reported seeking urgent care for conditions that they thought warranted medical care, but not emergency care. In some of these cases, the urgent care provider encouraged going to the ED.	I had a wound at the bottom of my good that was seeping…the leg kept getting redder…the redness was up to my knee…'Okay, I'm going to urgent care.’…The doctor looked at it and said,’…you really need to go to the ED. (English speaker)
Call 911	Call emergency medical services was not a predominant category. Participants would only call 911 if their condition quickly deteriorated but were otherwise ‘against ambulances’. One barrier to calling an ambulance was transportation fees. When emergency medical services is called, however, it provides a provider evaluation to inform patients’ decisions to seek ED care.	I started to realize something wasn't right and whatever was wrong was getting worse…I was aware of my surroundings, but for some reason I couldn't function…I called 911 through my videophone. At that time, I realized that my speech was also abnormal. I had a difficult time trying to communication with the 911 dispatcher. (ASL‐user)
Go to the ED immediately	Participants who chose to go immediately to the ED articulated the complexity of their decision: seeking social network advice, looking at their previous ED experiences, perceived consequences of not seeking care, access to transportation and perceived technical quality of care.	I was apprehensive about going to [the small] ER, knowing that they can't do an MRI. And they're really lax about trying to figure out what's going on…It's all about how much pain I'm in. If I'm to the point where I'm just fed up and just don't want to wait too long anymore, I'll take the 50‐50 change [that care will be bad] and I'll go [to the small ER]. (English speaker)

Abbreviations: ASL, American Sign Language; DHH, deaf and hard of hearing; ED, emergency department; ER, emergency room; MRI, magnetic resonance imaging.

### 3.2 Theme 2: Patient‐centred ED care differs between DHH ASL‐users and DHH English speakers

#### 3.2.1 Patients feeling stereotyped

DHH patients described situations of feeling stereotyped or judged by their ED care team, diminishing the trust and working alliance with providers. For DHH ASL‐users, their deafness was described as the basis of stereotyping.

[I'm concerned about] the physician's attitude. Whether or not they will patronize me. Whether their previous encounters with Deaf patients will dictate how they treat me or whether they see me as an individual. Whether they recognize that I am a patient with [multiple organ] failure and that my needs are unique. That I'm not just some other Deaf patient improperly taking advantage of the ER…time is wasted while they make the realization that my case isn't like the others. (ASL‐user)

This was partially attributed to the patients' belief that other DHH ASL‐users were accessing the ED for nonemergent concerns and that they would not be seen as unique patient.

DHH English‐speaking patients described similar concerns with being judged and stereotyped, but believed that these stereotypes were not related to being DHH. One patient with multiple disabilities, including a neurological disability, recounted:

I think they [providers] assume a position where they are intellectually superior to me. They talk to me like I am [age‐redacted] years old and I have dementia, and I can't think clearly…I think some physicians profile patients. They put patients in different categories – they stereotype a patient – and they attribute certain qualities and attributes to that patient that are true to not true. (English speaker)

Another DHH English speaker with a mobility disability recognized that ED workers are likely to be ‘stretched too thin’ but wished that staff were more compassionate, particularly in understanding the complexity of their disabilities.

#### 3.2.2 Being involved in the care process

DHH English speakers described the technical quality of care and addressing the precipitating condition as the top concerns in the ED, while DHH ASL‐users described communication access and ED staff's bedside manner as their top concerns. DHH English speakers described having ‘no trouble communicating’ with their care team.

Yes, they explained everything – they explained everything to me. They did a CT scan and they explained that they did that because I hit my head when I fell. And they wanted to make sure nothing was wrong with my head. And they did an EKG and the echo to look further into my heart. (English speaker)

DHH English speakers mentioned that they sometimes felt that providers were disrespectful; these experiences were reported more frequently by their ASL‐using counterparts. Further, the positive communication experiences discussed by DHH English speakers were strongly juxtaposed to the experiences reported by DHH ASL‐users. For example, where DHH English speakers could describe the rationale behind their treatment plans in detail, DHH ASL‐users reported the opposite.

They seemed to be fine with just trying different medicines on me without first figuring out why I was going through this. My blood pressure was still so high, and they couldn't give me anything more for it…I don't think there was any process or decisions it seemed. They just sort of told me what was happening next: x‐rays, meds, EKG, etc. …I just assume the doctors know what they are doing, and I just follow what they say. (ASL‐user)

However, both patient groups reported a preference for providers to use less medical terminology, particularly when using written communication.

#### 3.2.3 Pain communication

The general concerns discussed in understanding treatment and diagnosis decisions were also prevalent with respect to pain communication—particularly with the differences between patient groups. DHH English speakers, particularly those with higher health literacy and knowledge of medical terminology, described communicating their pain more easily than DHH ASL‐users.

I understand the difference between what I consider normal pain, where you would take an aspirin or something like that, and neuropathic pain. So, I'm able to tell the physician or the medical provider, I can be very specific as to the pain, the pain location, and the intensity. (English speaker)

Two DHH ASL‐users reported being shown the visual pain scale; the other two reported communicating through VRI or using their voice. The use of the visual pain scale was reported as beneficial to the communication process, allowing the DHH patients to ‘determine how [they] feel and match it to the faces on the paper’ (ASL‐user). There was concern, however, that the use of the DVPRS may impact appropriate pain management.

When I was shown the pain scale, I would point to just beyond the page to show that my pain was at 11 out of 10…sometimes I wonder if they view me differently than how they may view someone [who is not deaf]. I've described the agony before, but I can't help but wonder if they are really getting the same message that I'm conveying to them. I express that I'm in terrible pain, and yet sometimes it doesn't seem to sink in for them just how serious it is.

Interviewer: Do you feel like you can't explain your pain clearly to them because of the lack of an interpreter?

Right. The nurses sometimes just don't understand. I try and explain, but something gets lost in their cognitive process when they see me signing or gesturing, trying to describe the pain. (ASL‐user)

In this case, the DHH patient was concerned that the provider was misinterpreting or discounting their pain rating reported through the DVPRS tool. However, in member check interviews, a community advisory group member indicated additional concerns with the DVPRS, indicating that the facial expressions need to be more dramatic to fully communicate the seriousness of the pain.

#### 3.2.4 Accommodations and patient‐activated interpersonal and environmental modifications

DHH English speakers did not describe requiring physical accommodations (e.g., an interpreter) for their communication access; however, one participant mentioned that they wished closed captions were available in the hospital. Despite not requiring auxiliary aids, DHH English speakers discussed changes in patient–provider communication that they needed. These interpersonal communication modifications included asking care team members to remove their mask; face the patient; and repeat information when asked.

The first time they're not looking at me when they talk, that's usually when I tell them, ‘Please look at me’. I'll say, ‘I'm hard of hearing. Please look at me. I can't understand you when you're away from me. So, please just turn around, and talk directly to me, and make eye contact’. (English speaker)

Although providers were described as generally respecting English‐speaking patients' requests for these modifications, some participants discussed that if they had to repeat their request multiple times, they would stop asking due to the power dynamic with providers.

Sometimes I feel like it is not polite [to keep asking], because I cannot…[silence] Sometimes, it is just they are the ones who knows. They're important. You look at them. They know what they're doing. (English speaker)

DHH ASL‐users also described requesting interpersonal and environmental modifications and also reported requiring an ASL/English interpreter to facilitate communication with providers. These participants reported, however, that on‐site services were unavailable and that VRI quality was frequently inadequate. The lack of effective accommodations for these patients led to patients self‐advocating for interpreter services and care team providers to write back and forth.

After I made the request for an interpreter, they seemed evasive. Sometimes they would write, sometimes they would come back with a Video Remote Interpreting device, sometimes they would just talk at me and hope I could lipread it, to no avail. I had asked for an interpreter, but they took it upon themselves to experiment with many other communication methods before they conceded to my request, which should have been honored by default in the first place. (ASL‐user)

DHH ASL‐users' ability and willingness to self‐advocate for communication access were contingent on their condition, including pain, and the presence of others to support them in their self‐advocacy. DHH ASL‐users who attempted to self‐advocate using their spoken language skills reported additional issues of patient‐centredness stemming from the care team members’ misconceptions about DHH people.

I eventually got a nurse's attention and asked for an interpreter, however the nurse said that we were communicating just fine. I told her that with the masks that I just couldn't understand. She brushed me off, saying I spoke fine and decided that I could understand fine. (ASL‐user)

#### 3.2.5 Discharge processes and postdischarge information needs

The stark differences between DHH ASL‐users and English speakers remained when discussing the accessibility of discharge instructions and how that accessibility impacted postdischarge information needs.

DHH English speakers indicated that communication during discharge was accessible to them, but some thought that it was ‘too long’ or ‘too understandable’ (simple). These patients felt that their information needs were met and that they had sufficient understanding of the next steps of treatment (e.g., being referred to a specialty provider). Despite their information needs being met, there was anxiety about missing information.

Yes [it is very clear], and I also made it a point, ‘Oh my god! I'm going to have to read through it all word‐for‐word’ because being hard of hearing. It's, you know, it's possible to miss a sentence here and there. (English speaker)

In comparison, DHH ASL‐users indicated that ED discharge instructions and communication were not accessible to them.

I heard nothing from them until they were ready to discharge me. I do not know the reasoning behind my discharge. I had gone from having the episode at work, to the hospital and discharge, and still had no more information than when it happened. They simply said that I look fine, so it was time for me to go home. For the next few days, I was still dealing with dizziness and other symptoms. (ASL‐user)

In this participant's case, not only did they receive inadequate communication during the treatment and diagnosis process but they also did not receive adequate discharge instructions, which left them confused when their symptoms remanifested.

Diagnoses not being communicated to patients were common among DHH ASL‐users; this was attributed to the lack of communication access through an interpreter. In the rare occurrence when discharge occurred with an interpreter present (whether on‐site or through quality VRI services), DHH ASL‐users indicated increased engagement with providers.

During the discharge, I learned that I had pneumonia. I was able to ask, through the VRI, what caused the pneumonia, and also received instructions for filling my prescription and follow‐up in with my primary doctor. (ASL‐user)

Overall, DHH ASL‐users reported wanting more accessible instructions and more information about their condition. When discharge information was not accessible, DHH ASL‐users described calling medical providers using the Video Relay Service and searching the internet for images and websites focused on their condition. Comfort reading websites about their condition was dependent on their English proficiency, with one DHH ASL‐user citing receiving information from the CDC, NIH and Medline, and another DHH ASL‐user searching websites tailored for non‐DHH children. However, this is not patients' preferred route of receiving information.

I would prefer getting [discharge] information and education at the same time [at the ER], like hearing patients. I want to learn about my health through an on‐site interpreter. VRI does not work 100% of the time. Getting this information directly from the doctor is important because I don't know if I can fully trust the information I find online. Having the doctor explain what to do with an interpreter would be best, in addition to having videos and information available in ASL. (ASL‐user)

## 4 DISCUSSION

This study reports findings from a qualitative aim of an explanatory sequential mixed‐methods study. Quantitative outcomes of the parent study focused on ED utilization^26^ and on condition acuity, triage pain ratings, ED LOS and acute ED revisits.^28^ Our qualitative data contextualize these results. To facilitate a mixed‐methods integrated understanding, we used a joint display (Table 3) and narrative weaving to identify how qualitative findings explain quantitative results.^39^, ^51^

**Table 3:** Joint display explaining emergency department utilization and patient‐centred care perspectives among deaf and hard‐of‐hearing patients.

Quantitative results^26^, ^28^	Qualitative findings
DHH ASL‐users and DHH English speakers have higher adjusted odds than non‐DHH English speakers of using the ED in the past 36 months (aOR = 1.79 and 1.64, respectively).^26^ Among ED users, DHH ASL‐users and English speakers also use the ED more frequently than non‐DHH English speakers (70% and 61% more, respectively).^26^	As described in theme 1, DHH patients use the ED after engaging in a complex decision‐making process and considering their symptoms, health literacy, access to alternative sources of care and other social circumstances.
Condition acuity: Compared to encounters of non‐DHH English speakers, there were no significant differences among encounters of DHH ASL‐users (aOR = 1.07; 95% CI: 0.67–1.71) and DHH English speakers (aOR = 1.39; 95% CI: 0.99–1.95) being classified as high acuity.^28^	As described in Theme 1 (Evaluating Need), DHH ASL‐users and DHH English speakers report seeking healthcare for issues that they perceive as ‘serious’. Reasons for seeking ED care, mentioned in interviews, included the following: Diagnosed chronic health issues (e.g., heart failure). Undiagnosed chronic health issues (e.g., postural orthostatic tachycardia syndrome). Experiencing conditions for which medical providers encouraged seeking ED care.
Pain rating: Compared to non‐DHH English speakers, there were no mean differences in triage pain rating scores among DHH ASL‐users (*B* = 0.51, *p* = .30) and DHH English speakers (*B* = −0.19, *p* = .22).^28^	As described in theme 2 (Pain Communication), the communication context in the ED influences pain communication and perceived pain management. DHH English speakers report no issues with pain communication and report pain using spoken language. DHH ASL‐users report pain using the DVPRS pain scale, gestures, writing back and forth, interpreters and speaking for themselves. DHH ASL‐users are concerned that they receive inadequate pain management due to communication differences.
ED length of stay: DHH ASL‐users had, on average, longer ED LOS (by 30 min) compared to non‐DHH English speakers. There was no difference in ED LOS when comparing DHH and non‐DHH English speakers.^28^	In interviews, DHH participants discussed waiting in the ED as a general marker of the quality of care, with longer encounters described as a ‘waste of time’ due to ED burden. DHH ASL‐users reported waiting for effective communication access – either through on‐site interpreters or VRI.
Acute revisit: DHH English speakers represented a majority of acute revisit encounters (61%), while DHH ASL‐users represented less than one‐fifth (16%).^28^	As described in theme 2 (Discharge Processes and Postdischarge Information Needs), there are differences in the experiences of DHH ASL‐users and DHH English speakers at discharge. DHH English‐speaking patients receive high‐quality ED discharge. DHH ASL‐users, however, describe situations of ED discharge failure. The negative healthcare experiences of DHH ASL‐users lead them to disengage from future ED care‐seeking, even when symptoms may necessitate use.

Abbreviations: aOR, adjusted odds ratio; ASL, American Sign Language; CI, confidence interval; DHH, deaf and hard of hearing; DVPRS: Defense and Veterans Pain Rating Scale; ED, emergency department; LOS, length of stay; VRI, video remote interpreting.

### 4.1 ED utilization

Multiple studies, including one from the parent mixed‐methods study,^26^ suggest that DHH patients are at higher risk of ED utilization than non‐DHH people.^6^, ^27^, ^29^ There were no differences between DHH ASL‐users and English speakers when compared to non‐DHH English speakers with respect to discharge diagnoses^26^ or ED triage condition acuity level (using the Emergency Severity Index^52^).^28^ Based on findings from this qualitative study, DHH patients report seeking ED care for issues that they perceive as serious, including diagnosed and undiagnosed chronic health issues (e.g., cardiovascular diseases and disorders), or in circumstances when healthcare providers recommended seeking care. Based on the synthesis of participants' experiences and reasons for seeking the ED, DHH patients seek ED care for rational and justified reasons based on their circumstances. This supports the critique of Andersen's conceptualization of ‘patient perceived need’^53^ in the *Conceptual Model of ED Utilization among DHH Patients* ^11^; DHH patients *are* evaluating their need and ability to engage in ED care, with respect to health status, and patient‐ and nonpatient factors.

### 4.2 ED care

Once in the ED, DHH patients' experiences diverge based on their preferred language modality and how they perceive providers response to these modalities. A discussion regarding the practice, social and legal context surrounding communication access in EDs is beyond the scope of this paper, but inaccessible patient–provider communication—particularly among DHH ASL‐users—is more prominent than not.^16^, ^19^, ^20^, ^21^

#### 4.2.1 Pain

Although there were no differences in triage pain scale reports,^28^ pain communication was a central category influencing DHH patients' care experiences. DHH English speakers reported communicating their pain using spoken English, and DHH ASL‐users reported pain through the visual pain scale tool, gestures, writing back and forth, spoken English and, infrequently,^21^ through interpreters. DHH English speakers reported positive experiences with pain communication and management and were able to leverage higher self‐reported health literacy to describe the intensity, location and history of the pain. DHH ASL‐users, however, reported poorer pain management and felt that communication barriers were restrictive to their pain communication. Specifically, it is possible that ASL‐using patients in this study misunderstood how to use the pain scale and that reporting a 10 is the worst pain that the patient could ever experience. This is noteworthy because the DVPRS uses facial expressions^54^—which typically aids limited English‐speaking populations in communicating pain^55^—which are a natural feature of ASL. Therefore, additional research is needed to understand the cultural relevance and understanding of the DVPRS (and similar pain scale tools) among this population.

#### 4.2.2 ED LOS

DHH ASL‐users also had longer ED LOS than non‐DHH English speakers: 30 min longer, on average.^28^ LOS and wait times are a common occurrence for all patients in the ED; therefore, wait times were not a focus of the qualitative analysis. However, in interviews, DHH patients recognized that ED burden and facility location influenced wait times and stated that longer stays were a ‘waste of time’. Longer LOS for DHH ASL‐users was attributed to a lack of effective communication and being unable to discuss their condition history with providers, and long wait times for on‐site interpreters or VRI. This is supported by other research on DHH patient ED utilization, where some patients report waiting up to 8 h for an onsite interpreter.^21^

#### 4.2.3 Discharge

We hypothesized that DHH ASL‐users would represent a majority of acute ED revisits. Contrary to this hypothesis, results indicated that a majority of revisits were among DHH English speakers.^28^ This finding was unexpected, as DHH ASL‐users' ineffective ED communication experiences,^21^ particularly at discharge, may be classified as ED discharge failure.^56^ In the present study, DHH English‐speaking patients reported that care coordination (e.g., referral to a specialist provider) and ED discharge communication met their information needs. Based on the qualitative interviews, ED discharges for DHH English‐speaking patients were high quality and did not suggest discharge failure. However, the discharge experiences of DHH ASL‐users were indicative of discharge failure based on poor health information comprehension.

One potential explanation is the form of the relation between ED discharge failure and acute revisit. A high‐quality discharge is thought to decrease the occurrence of ED revisits, while ED discharge failure increases the probability of a revisit.^56^, ^57^ However, qualitative findings indicate that DHH ASL‐users may disengage from ED care‐seeking due to poor patient experiences even when they should reasonably re‐seek ED care. Given the qualitative, and socio‐medical, differences between DHH ASL‐users and pre‐ and postlingually DHH English speakers,^11^ future research should seek to further contextualize the reasons for which DHH patients revisit the ED.

### 4.3 Recommendations for ED care

Participants in our sample indicated that they wanted providers to be more compassionate to their circumstances, less judgemental and have more content knowledge of multiple disabilities to assist with providing patient‐centred care. The use of electronic health record systems (e.g., alerts or notifications) may assist providers with more directly centring patients' experiences and accessibility needs.^58^, ^59^ Mirroring research regarding limited disability education in undergraduate and graduate medical education,^60^, ^61^, ^62^ both DHH ASL‐users and English speakers recognized that health professional preparation programmes had ‘shortcomings’ and ‘failings’ that directly impact patients with disabilities. Participants encouraged the inclusion of disability content across the curriculum and ensured that students had the opportunity to interact with a variety of patients with disabilities.^62^

Communication in the ED, like other healthcare settings, must be accessible to DHH patients. Writing back and forth, the use of friends or family as communication facilitators or relying solely on one‐way communication tools (e.g., visual pain scales) is largely ineffective and dangerous for DHH patients as these methods of communication are fraught with opportunities for miscommunication.^21^ ED care staff must become familiar with their organization's communication access plans and advocate for DHH patients’ accommodation requests to be fulfilled.

Participants in the sample—particularly DHH ASL‐users—would prefer additional information from ED providers on their health status and treatment plans. The lack of accessible education at discharge, however, left patients wanting more information and then having to navigate inaccessible (and potentially unreliable) online health resources. Therefore, discharge instructions and education should be provided in the patient's preferred modality, and more web‐based DHH‐friendly health resources should be developed and disseminated. The electronic medical record should be leveraged to incorporate informative ASL discharge videos relating to the patient's discharge diagnoses including homecare instructions and return precautions. For example, videos were created by community‐ and academic‐based partners in Rochester, NY, to improve discharge quality.^63^

The limitations and strengths of this study should be considered when evaluating results. First, there was a small sample size relative to most other qualitative work and, consistent with the qualitative tradition, we do not promote the claim of generalizability of these results. Consistent with qualitative methodology,^46^, ^47^, ^64^ the length of both original and member check interviews, the interviewer's prolonged engagement with the community, knowledge of DHH culture and established trust with the local community lend more credibility to the data.^45^ Considering the richness of interview data and appropriateness of the analytic technique, we are confident that the findings of this study are trustworthy and represent these DHH patients' experiences in the local context (i.e., in north central Florida). Two additional methods to increase credibility included peer debriefing and conducting member check interviews with interviewees and community advisory group members. Further, to facilitate transferability to other settings, we have provided a rich description and represented quotes throughout.^45^, ^65^ A limitation, however, is the lack of triangulation with ED provider perspectives; these perspectives would serve as a source of triangulation, allowing the field to better understand the potential influence of ED infrastructure on DHH patient care.

## 5 CONCLUSION

This article reports findings from a mixed‐methods study to better explain DHH patient ED utilization. The mixed‐methods integrated findings support the notion that DHH patients engage in a complex, decision‐making process when seeking ED care. Our qualitative findings—when evaluated holistically and analysed through the lens of ableism and audism—suggest that these and other root causes of oppression influence multiple steps of the ED care‐seeking and care process. Further, the poorer patient experiences reported by DHH ASL‐users may be partly attributed to these patients being perceived as less socially normative than DHH English speakers who are using the majority language. These findings have direct implications for the provision of patient‐centred ED care and patient health education.

## AUTHOR CONTRIBUTIONS

**Tyler G. James**: Conceptualization; methodology; formal analysis; investigation; writing—original draft; writing—revision and editing and funding acquisition. **Meagan K. Sullivan**: Conceptualization; validation; investigation; writing—original draft and writing—revision and editing; **Michael M. McKee**: Conceptualization; methodology; validation and resources. **Jason Rotoli**: Conceptualization; and writing—revision and editing. **David Maruca**: Conceptualization; resources; validation and writing—revision and editing. **Rosemarie Stachowiak**: Investigation; validation; writing—revision and editing. **JeeWon Cheong**: Writing—revision and editing and supervision; **Jason Rotoli**: Methodology; validation; writing—revision and editing and supervision.

## CONFLICT OF INTEREST STATEMENT

The authors declare no conflict of interest.

## ETHICS STATEMENT

This study was approved by the University of Florida Institutional Review Board (IRB#201903489). Participants provided consent before the study. Research data are not shared.

## Supporting information

Supporting information.

## Data Availability

The data that support the findings of this study are not available as participants did not consent to full transcripts being shared.
