# Supplementary material for: Emergency department patient‐centred care perspectives from deaf and hard‐of‐hearing patients
Source: Health Expect. 2023 Aug 9;26(6):2374–86. doi: 10.1111/hex.13842 (PMC10632638; doi:10.1111/hex.13842)
Supplement: Supplementary file 1 — Supporting information. [file HEX-26--s001.docx]

**Article:** Emergency Department Patient-Centered Care Perspectives from Deaf and Hard-of-Hearing Patients

**Supplemental Material 1:** Interview guide

**SEMI-STRUCTURED INTERVIEW:**

*Explain to the participant that we are going to begin asking questions related to their experience in the emergency room over the past 3 years, specifically focused on their time in the ER at [location]. Emphasize that their honest answers will be most helpful and that their information is confidential.*

Note to IRB: These questions and prompts are based on results from the quantitative aim of this study (using an explanatory sequential mixed methods design). Depending on how the participant elaborates each question, the interviewer will have to remain flexible. Due to the semi-structured, open-ended, conversational nature of the interviews, probes will be used based on participants' responses to further explore their answers **in-depth** after asking a broad open-ended question. Some probes are pre-determined, and they are listed below. Other probes will materialize as a result of what the participant shares. However, all probes and questions will be broadly informed by the following questions. Broad, open-ended guiding questions for the interviews will be:

1. Tell me about a time that you recently went to the emergency room at [location].
   **Probes to explore:**

- Deciding to use the ER
  - How did you decide you needed to go to the emergency room? (How did you assess the severity of your symptoms?) [Patient evaluated need]
  - Did you contact friends or family members for help with the decision? [Reinforcing: Family and social network factors]
  - Did you contact your primary care provider for an appointment before going to the ER? [Provider evaluated need]
  - How long did it take you to decide to use the ER? [Patient evaluated need and decision making]
- How/do you disclose to your medical providers that you are DHH?
- ED care processes
  - When in the ER, how do you communicate your level of pain to your providers?
  - [Prompt using DVPRS, see page 7] Have you seen this image when you go to the ER?
  - When going through diagnostic and treatment decisions with your doctor, how engaged do you feel?
- ED discharge
  - How would you describe the quality of communication with providers during discharge from the ER? [ED communication context]
  - How clear were the discharge instructions? Were they easy to understand? [Discharge]

1. Have you ever left the emergency room, only to go back for the same concern a few days later? Can you tell me what happened?

**Probes to explore:**

- How do you decide that you need to go back to the emergency room? [ED revisit]

1. Has there been a time that you thought about going to the emergency room, but decided not to?

**Probes to explore:**

- Role of social networks
- Patient evaluated need

1. Invitation to share other perspectives or information not covered during the interview.
